# Scoring System for Tumor-Infiltrating Lymphocytes and Its Prognostic Value for Gastric Cancer

**DOI:** 10.3389/fimmu.2019.00071

**Published:** 2019-01-29

**Authors:** Dachuan Zhang, Wenting He, Chao Wu, Yan Tan, Yang He, Bin Xu, Lujun Chen, Qing Li, Jingting Jiang

**Affiliations:** ^1^Department of Pathology, The Third Affiliated Hospital of Soochow University, Changzhou, China; ^2^Department of Tumor Biological Treatment, The Third Affiliated Hospital of Soochow University, Changzhou, China; ^3^Department of Oncology, The Third Affiliated Hospital of Soochow University, Changzhou, China; ^4^Jiangsu Engineering Research Center for Tumor Immunotherapy, Changzhou, China; ^5^Institute of Cell Therapy Soochow University, Changzhou, China

**Keywords:** tumor-infiltrating lymphocytes, gastric cancer, prognosis, CD3, tumor microenvironment

## Abstract

The tumor microenvironment (TME) is the internal environment of malignant tumor progression, and the host antitumor immune response and normal tissue destruction occur in the TME. Tumor-infiltrating lymphocytes (TIL) is a crucial component of the TME and reflect the host antitumor immune response. The purpose of this study was to discuss the methodology for TIL evaluation and assess the prognostic value of TIL in gastric cancer. In total, we reviewed 1,033 gastrectomy cases between 2002 and 2008 at the Third Affiliated Hospital of Soochow University. To understand the prognostic value of TIL in gastric cancer (GC), TIL were assessed by optical microscopy, and verified by immunohistochemistry. There is no current consensus on TIL scoring in GC. In this study, we discussed a TIL evaluation system that includes an analysis of the amount and percentage of TIL in a tumor. Ultimately, 439 (52.7%) cases showed high levels of TIL and 394 (47.3%) cases had low levels. There was a statistically significant relationship among TIL, tumor size, histological grade, LN metastasis, nerve invasion, tumor thrombus, pTN stage, and WHO subtypes (*p* < 0.001, respectively). TIL^hi^ was a positive significant predictor of overall survival (OS) in Kaplan–Meier survival analysis (*P* < 0.001) and multivariate Cox regression analysis (*HR* = 0.431, 95% CI: 0.347–0.534, *P* < 0.001). After surgery, patients with malignant tumors underwent chemoradiotherapy according to standard therapeutic guidelines based on TNM stage. The TNM scoring system cannot reflect the full information of TME; therefore, TIL can be used as a diagnostic supplement. We constructed a nomogram model that showed more predictive accuracy for OS than pTN stage. In summary, this study proves that high levels of TIL are associated with a positive prognosis and that TIL reflect the protective host antitumor immune response.

## Introduction

The tumor microenvironment (TME) is the internal environment of malignant tumor progression, and the host antitumor immune response and normal tissue destruction occur in the TME (1). Thus, the TME is emerging as a crucial factor for understanding the relationship between the immune system and tumor (2, 3). Tumor-infiltrating lymphocyte (TIL) is an important component of the TME and reflects the host antitumor immune response (4–6). In some solid tumors, such as ovarian cancer, breast cancer, and colorectal cancer, TIL are crucial for inhibiting cancer progression and have implications for the success of active cancer immunotherapy (7–9). The accumulating evidence form several researches indicates that TIL is predictive for response to neoadjuvant therapy and adjuvant chemotherapy for breast cancer patients (10, 11). Several studies in gastric cancer suggested that TIL and its components may direct patient selection for immunotherapy and checkpoint blockade therapy (12, 13). This recommends that the TIL evaluation in daily pathological diagnosis has become more important. In particular, the quantitative expression analysis of immune gene was typically high correlation with TIL (14), suggesting that TIL evaluation may be a valid, less expensive, and readily available alternative (15). Unlike breast cancer (16), there is no current consensus on the morphologic evaluation of TIL in GC. The objective of this study aimed to discuss the methodology for the morphologic TIL evaluation and assess TIL scoring in a cohort of 1,033 cases by OS and provide basic data for international TIL scoring of GC.

## Materials and Methods

### Patient and Tissue Samples

We conducted a retrospective cohort study using data from the Department of Pathology, Third Affiliated Hospital of Soochow University, from 2002 to 2008. The patients were enrolled according to the following criteria: (1) pathologically diagnosed with primary gastric adenocarcinoma; (2) naive to preoperative chemotherapy or radiotherapy; (3) adequate formalin-fixed and paraffin-embedded (FFPE) tissue blocks; (4) at least one slide containing the tumor invasive margin; (5) complete medical records and follow-up information. Ultimately, a total of 1,033 GC patient were included in this study. Two or three sections of cancer tissue were obtained from each patient and a total of 2,858 slides were reviewed. To construct and validate the analyses for TIL, 200 patients from 2005 to 2006 were enrolled in the external validation cohort, while the other patients (833 cases) were included in the primary cohort. The pathologic staging system was based on the seventh edition of the Union for International Cancer Control/American Joint Committee on Cancer (UICC/AJCC) for GC. The patients' survival intervals were available and dated to the end of November 2011. The study protocol was performed under the guidelines outlined in the Declaration of Helsinki and was approved by the Ethics Committee of Soochow University.

### Procedures

Unlike breast cancer (16), there is no current consensus or international guidelines on the morphologic evaluation of TIL in GC. The methodology for the TIL scoring system by Denkert et al. (11, 17) and the International TILs Working Group (16) was based on the hematoxylin and eosin (H&E) slides in breast cancer. In addition, the methodology for the TIL scoring system in another study conducted by Galon et al. (18–20) was based on the immunohistochemistry in colorectal cancer. This present research aimed to discuss the methodology for a TIL scoring system in GC. TIL evaluation was retrospectively done using H&E slides.

**Figure 1:**
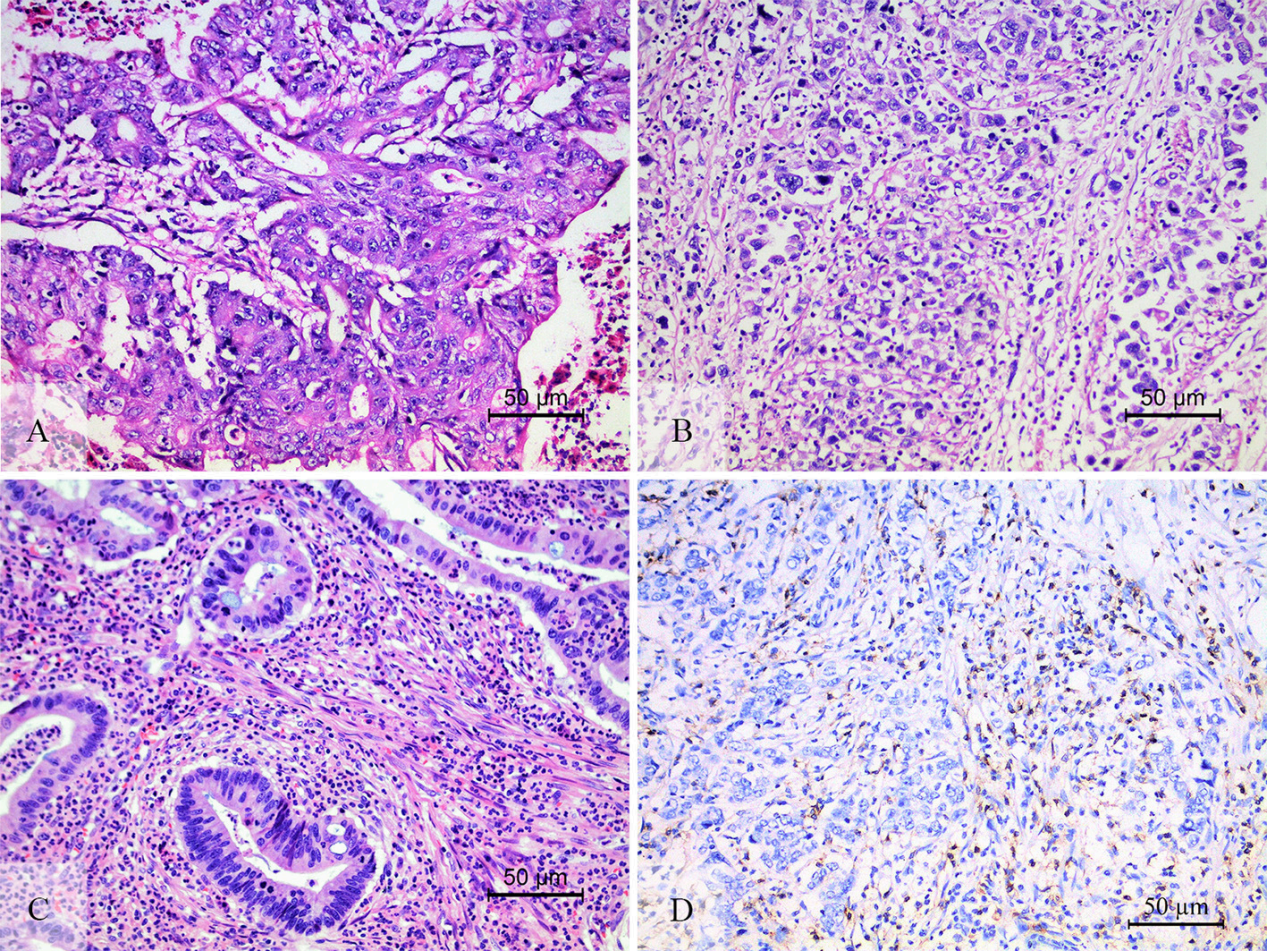
The level of TIL and CD3^+^ TIL. The low level of TIL **(A)**, showed the mild increase of infiltrating lymphocytes in the tumor nest and stroma. The high level of TIL **(B,C)**. **(B)** Showed that increased intratumoral TIL was interwoven with tumor tissue. **(C)** Showed that prominent stromal TIL was incorporated in tumor stromal. CD3 staining was performed to check the accuracy of morphologic TIL evaluation **(D)**.

This study adopted and modified the TIL scoring system used in previous studies. At first, the tumor area was divided into the center of the tumor (CT) and the invasive margin (IM) according to the criterion in the studies of Galon et al. (18) and Klintrup (21). The IM was defined as the junctional area between the tumor invading edge area and the host stroma (Supplemental Figure 1). The TIL evaluation was conducted operated separately in these two regions, and the two features of TIL intensity (21–23) and percentage (16, 22, 24) in the center, and invasive margin of the tumor were incorporated. All available complete slides were morphologically analyzed for (i) the intensity of TIL (score 0, no infiltrating lymphocytes; score 1, mild increase of infiltrating lymphocytes in the tumor nest or stroma; score 2, increased infiltrating lymphocytes interwoven with tumor tissue; score 3, prominent infiltrating lymphocytes separate or incorporated in tumor tissue) (Figure 1); (ii) the percentage of CT or IM region infiltrated by TIL in 10% increments (if <10% of the CT or IM was infiltrated by TIL, 1 or 5% criterium was used). TIL assessment was done independently by two pathologists who were blinded to the clinical data. During subsequent scoring, any problematic cases were discussed with two pathologists. After TIL assessment, we had a series of TIL scores: (i) score 1, the intensity (amount) of TIL in the CT; (ii) score 2, the intensity (amount) of TIL in the IM; (iii) score 3, the TIL-ct region score that reflect the distribution and density of TIL in the CT (score 1 × the percentage of the CT region infiltrated by TIL); (iv) score 4, the TIL-im region score that reflect the distribution and density of TIL in the IM (score 2 × the percentage of the IM region infiltrated by TIL); (v) score 5, the TIL-total score in the CT and IM (the sum of score 3 and score 4).

In order to verify the repeatability and accuracy of the TIL scoring system, we double checked it. First, 200 randomly selected cases in primary cohort were assessed using the scoring system described by Denkert et al. (11, 14) and Kang et al. (23). The methodology of these scoring system was used to assess the intratumoral TIL (iTu-TIL) and stromal TIL (str-TIL). The iTu-TIL and str-TIL were assessed by four pathologists and used for the statistical analysis. During subsequent scoring, any problematic cases were discussed with four pathologists. Second, 63 randomly selected cases were immunohistochemically stained with CD3 to verify the accuracy of the TIL scoring system. Immunohistochemical staining of CD3 positive expression in the cytoplasm and cell membrane was considered as positive. The score of CD3^+^ TIL is the number of positive cells under five high-magnification fields (400×) obtained randomly in the CT or IM.

### Statistical Analysis

The analysis was carried out using the IBM SPSS statistical software V24 (IBM, Armonk, NY), GraphPad Prism 6 (GraphPad Software, La Jolla, CA) and R software V3.4.3 (http://www.r-project.org/) with the foreign, rms, and pROC packages. All statistical analyses were two-sided tests and the statistical significance was defined as *P* < 0.05. The intraclass correlation coefficient (ICC) was used to evaluation the reproducibility of different pathologists (15, 25). The ICC for single measures was calculated using the mixed model and absolute agreement. A time-dependent receiver operating characteristic (ROC) curve analysis was performed to calculate the area under the curve (AUC) in order to compare the discriminatory power for the patients' overall survival (OS) between different TIL scores. Moreover, the ROC curve analysis was used to select the cut-off value (26, 27). To separate high and low level of TIL, the criterion for selection of cut-off point was the maximum of Youden index, which was defined as max_*c*_ [Sen(*c*) + Spe(*c*) − 1], where *c* is the cut-off point (score5: cut-off value 0.55, AUC 0.728, sensitivity 70.84%, specificity 67.43%; CD3^+^ TIL: cut-off value 830, AUC 0.647, sensitivity 79.49%, specificity 50.00%; score 5 in the primary cohort: cut-off value 0.55, AUC 0.693, sensitivity 72.15%, specificity 66.43%). The correlation between variables was examined using the Spearman correlation coefficient (R). The assessment of the association between TIL and clinicopathologic parameters was carried out using the χ^2^ test. Kaplan–Meier curves and the log-rank test were used for the survival analysis. To determine whether TIL are an independent prognostic factor for patient outcomes, univariate, and multivariate Cox proportional hazards regression models were constructed. The hazard ratio (HR) and its 95% confidence interval (CI) were evaluated for each factor. The χ^2^ test, Kaplan–Meier survival analysis, Cox proportional hazards regression model were performed in primary cohort and was validated in validation cohort. The nomogram model was formulated based on the results of the multivariate Cox regression analysis. A final model selection was performed using a backward stepdown selection process with the Akaike information criterion (AIC) (28). To evaluate the nomogram performance, the discrimination and calibration of the model were assessed. During the external validation of the nomogram, the total score for each patient in the validation cohort was calculated according to the generated nomogram. Then, Cox regression was performed in this cohort using the total score as a factor, and finally, the concordance index (C-index) and calibration curve were derived based on the regression analysis. Nomogram construction and validation were performed in accordance with the nomogram guide (29, 30).

## Results

### The Best Index of TIL

Before TIL assessment, two pathologists (DC and Qing) were trained to the TIL scoring system and 150 cases were assessed by them. The intraclass correlation coefficient (ICC) was used to evaluation the reproducibility of different pathologists (ICC 0.845, 95% CI: 0.790–0.886, *P* < 0.001). First, the analysis was based on the data of the primary cohort. We compared the AUC of TIL scores using ROC curve analysis and score 5 had the highest AUC 0.741 (Table 1, Supplemental Figure 2A). In the series of TIL scores, score 3, 4, and 5 were semiquantitative variables. To make the scoring system more facile and ease to the statistical analysis, variables were transformed as followed: (i) the original four-point scale (score 1 and 2) were reduced to a two-point scale: absent to mild increase (0, 1) were combined as low-level TIL and moderate to strong (2, 3) as high-level TIL; (ii) score 3, 4, and 5 were converted into binary variables according to cut-off value calculated by the ROC curve analysis (Supplemental Table 1). In the Kaplan–Meier survival analysis, patients with a high level of score 5 had the best prognosis (χ^2^ = 148.762, *P* < 0.001, Table 1). In the multivariate Cox regression analysis, score 5 was an independent prognostic factor (*HR* = 0.431, 95% CI: 0.347–0.534, *P* < 0.001, Table 1). Then, the validation cohort was used to validate, and the result was the same as primary cohort (Table 1, Supplemental Table 1). According to these analyses, score 5 (the TIL-total score) was chosen as the representative of the TIL evaluation and it was used for the followed statistical analysis.

**Table 1:** The comparation to a cohort of TIL score.

**A cohort of TIL scoring**	**Patients**	**Status**	**Mean OS**	**Log-rank**	***P-*value**	AUC^*^	Multivariate Cox analysis^**^		
	**(*n* = 833)**	**Death**	**(month)**	**χ^**2**^**			**HR**	**95% CI**	***P-*value**
**SCORE 1: THE INTENSITY OF TIL IN CT**									
High	259	52	49.04	121.755	**<0.001**	0.721	0.346	0.256–0.468	**<0.001**
Low	574	368	86.21						
**SCORE 2: THE INTENSITY OF TIL IN IM**									
High	254	80	52.16	58.334	**<0.001**	0.604	0.415	0.322–0.535	**<0.001**
Low	579	340	79.50						
**SCORE 3: THE TIL-ct REGION SCORE**									
High	352	97	45.89	130.646	**<0.001**	0.728	0.430	0.339–0.546	**<0.001**
Low	481	323	81.73						
**SCORE 4: THE TIL-im REGION SCORE**									
High	218	60	52.27	69.273	**<0.001**	0.608	0.352	0.265–0.468	**<0.001**
Low	615	360	83.61						
**SCORE 5: THE TIL-TOTAL SCORE**									
High	439	141	41.21	148.762	**<0.001**	0.741	0.431	0.347–0.534	**<0.001**
Low	394	279	78.17						

*Values in bold signify P < 0.05*.

* *The AUC was calculated by time-dependent ROC curve analysis using semi-quantitative variable*.

** *The detail of Multivariate Cox analysis was showed in the Supplemental Table 6*.

### The Repeatability and Accuracy of the Morphologic Evaluation of TIL

The iTu-TIL were assessed by two pathologists, Qing and Chao, (ICC 0.889, 95% CI: 0.855–0.915, *P* < 0.001) and str-TIL were assessed by two pathologists, DC and Yang, (ICC 0.934, 95% CI: 0.913–0.949, *P* < 0.001). The iTu- and str-TIL had the significant correlation with the TIL (*r* = 0.815, *P* < 0.001; *r* = 0.900, *P* < 0.001, Supplemental Figure 3). The median values of iTu- and str-TIL were then used as the cut-off values, and all cases were subdivided into positive and negative. The χ^2^ test and consistency analysis showed that iTu-TIL and TIL had significant agreement (χ^2^ = 79.58, *P* < 0.001, and κ = 0.630, *P* < 0.001, Supplemental Table 2). The same result was shown between str-TIL and TIL (χ^2^ = 131.8, *P* < 0.001 and κ = 0.809, *P* < 0.001, Supplemental Table 2). These results showed a high level of repeatability between these two TIL scoring systems and proved the morphologic evaluation of TIL that based on the H&E slides was reproducible. The ROC analysis showed TIL had the higher AUC (0.803) than str-TIL (0.738) or iTu-TIL (0.727) (Supplemental Figure 2B). This result suggested that TIL was a superior parameter than str-TIL or iTu-TIL.

TIL is an aggregation of multiple subtypes of lymphocytes, and the major component is CD3^+^ T cells (16, 18). Therefore, we chose CD3^+^ TIL to verify the accuracy of the morphologic evaluation of TIL (Figure 1D). The statistical correlation analysis showed a positive correlation between the TIL and CD3^+^ TIL (*r* = 0.691, *P* < 0.001, Supplemental Figure 3). We further grouped 63 patients into two groups according to the CD3^+^ TIL cut-off value (830) that was calculated by the ROC curve analysis: CD3^+^ TIL^low^ with 20 cases and CD3^+^ TIL^hi^ with 43 cases. The χ^2^ test and consistency analysis showed significant agreement between CD3^+^ TIL and TIL (χ^2^ = 27.94, *P* < 0.001 and κ = 0.666, *P* < 0.001, Supplemental Table 2).

### The Relationship Between TIL and Clinicopathologic Parameters

According to the TIL cut-off value (0.55) that was calculated by the ROC curve analysis, the primary cohort were divided into two groups: TIL^hi^ with 439 cases (52.7%) and TIL^low^ with 394 cases (47.3%) (Table 1). The details of the clinicopathologic parameters in the primary cohort, the validation cohort, and the complete cohort were shown in Supplemental Tables 3, 4. The TIL^hi^ was significantly correlated with small tumor size (*P* < 0.001), well-differentiation histological grade (*P* < 0.001), negative LN metastasis (*P* < 0.001), negative nerve invasion (*P* < 0.001), negative tumor thrombus (*P* = 0.003), early/low pTN stage (*P* < 0.001), WHO subtypes (*P* < 0.001), radical gastrectomy (*P* < 0.001, Table 2). In the validation cohort, the TIL^hi^ was also significantly correlated with well-differentiation histological grade, negative LN metastasis, negative tumor thrombus, early/low pTN stage, WHO subtypes, radical gastrectomy (Table 2). Clinicopathologic parameters are widely used to evaluate tumor malignancy and the prognosis of patients and to develop clinical oncology treatments. In this study, the data showed that TIL^hi^ was positively associated with most of the tumor clinicopathologic parameters. This relationship revealed that TIL represented an enhancement of the host antitumor immune response and a positive prognosis for GC patients. TIL can be a strong prognostic indicator and as crucial as the clinicopathologic parameters.

**Table 2:** The correlation between TIL and clinicopathological parameters.

**Clinicopathological parameters**	**TIL-primary**		**χ^2^**	***P-*value**	**TIL-validation**		**χ^2^**	***P-value***
	**High**	**Low**			**High**	**Low**		
**GENDER**								
Male	314	290	0.450	0.502	54	62	0.001	0.986
Female	125	104			39	45		
**AGE (YEAR)**								
≤ 50	74	53	1.863	0.172	8	14	1.021	0.312
>50	365	341			85	93		
**TUMOR SIZE (cm)**								
≤ 5	237	159	15.470	** < 0.001**	49	50	0.707	0.400
>5	202	235			44	57		
**HISTOLOGICAL GRADE**								
Well	29	9	42.482	** < 0.001**	8	5	14.694	**<0.001**
Moderately	191	103			51	34		
Poor	219	282			34	68		
**LN METASTASIS**								
Positive	221	299	57.773	**<0.001**	48	80	11.577	**<0.001**
Negative	218	95			45	27		
**NEURAL INVASION**								
Positive	169	244	45.608	**<0.001**	46	66	3.015	0.082
Negative	270	150			47	41		
**TUMOR THROMBUS**								
Positive	94	120	8.898	**0.003**	47	70	4.540	**0.033**
Negative	345	274			46	37		
**pTN STAGE**								
I	159	55	75.970	**<0.001**	33	16	16.923	**<0.001**
II	137	103			33	32		
III	143	236			27	59		
**WHO SUBTYPES**								
Tubular	323	203	54.597	**<0.001**	70	53	18.123	**<0.001**
Mucinous	24	57			2	15		
Papillary	26	19			4	7		
Poorly cohesive	51	78			9	17		
Undifferentiated	15	37			8	15	
**GASTRECTOMY**								
Radical	415	346	11.860	**0.001**	90	94	4.239	**0.040**
Palliative	24	48			3	13		
**CHEMOTHERAPY**								
Positive	159	168	3.590	0.058	35	50	1.684	0.194
Negative	280	226			58	57		

*Values in bold signify P < 0.05*.

### Prognostic Value of TIL in Gastric Cancer

**Figure 2:**
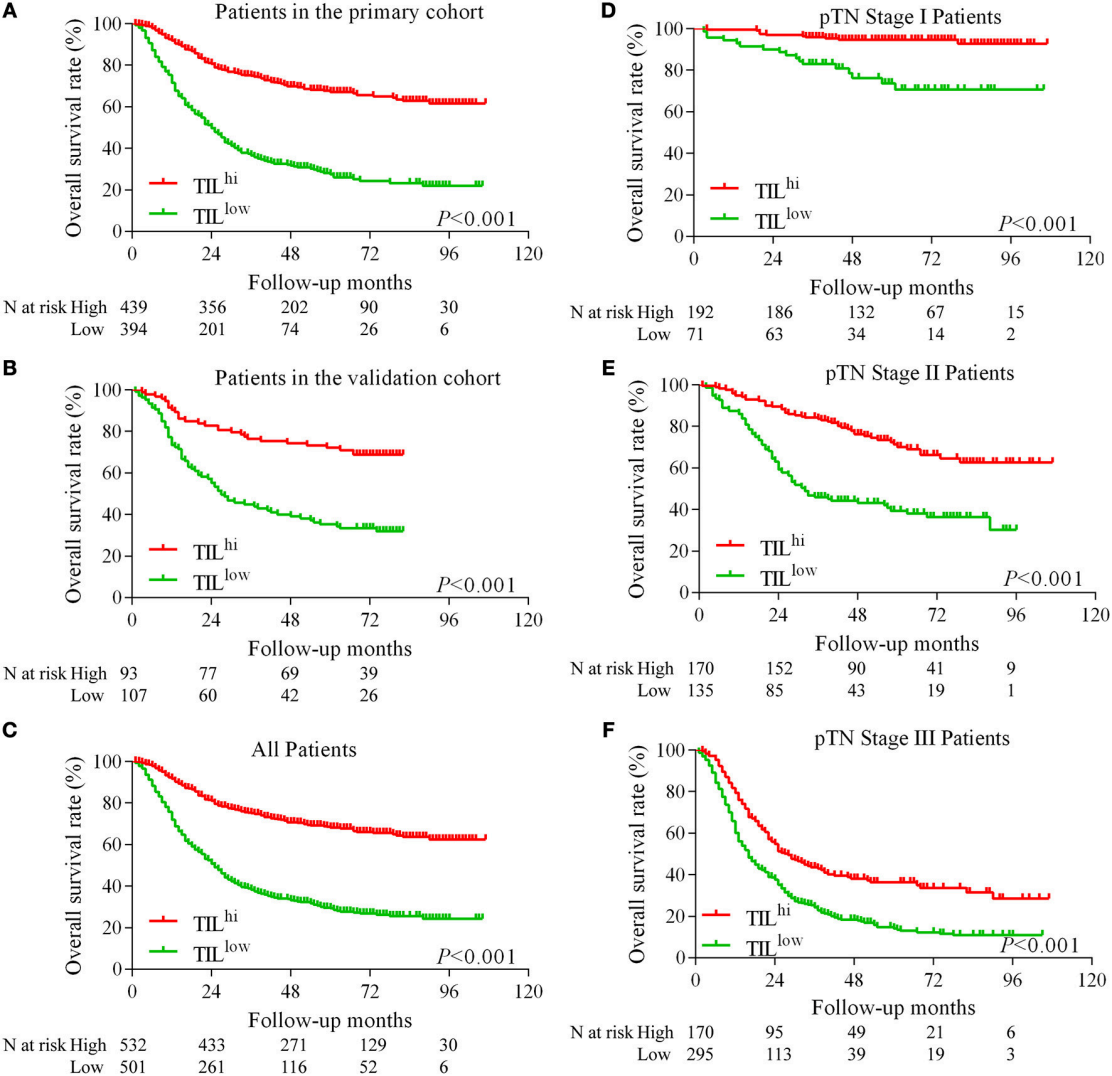
Kaplan–Meier survival curves for TIL and pTN stage in gastric cancer. Kaplan–Meier survival curves for the primary cohort, the validation cohort and the complete cohort **(A–C)**. Kaplan–Meier survival curves for pTN I-III stage as a function of TIL **(D–F)**.

The Kaplan-Meier survival analysis in primary cohort showed that the average survival period of patients with TIL^hi^ (78.17 ± 2.00 months) compared to that of patients with TIL^low^ (41.21 ± 2.09 months) was significantly different (χ^2^ = 148.762, *P* < 0.001, Table 1, Figure 2A). The data showed that patients with TIL^hi^ had a better prognosis than the TIL^low^ patients. The Kaplan–Meier survival analysis in validation cohort and the complete cohort showed that the patients with TIL^hi^ had better prognosis to the patients with TIL^low^ (χ^2^ = 28.857, *P* < 0.001, χ^2^ = 174.77, *P* < 0.001, Figures 2B,C). Moreover, the contribution of TIL to the prognostic power of each pTN stage was tested in the complete cohort. Low and high levels of TIL had significant prognostic value for pTN stage I-III patients, and the trend showed that the prognosis was better with TIL^hi^ (Figures 2D–F).

Clinicopathologic parameters were observed as an important indicator of a cancer patient's prognosis. Cox regression analysis was conducted to evaluate the prognostic significance of TIL and clinicopathologic parameters. The variables were age, tumor size, histological grade, LN metastasis, neural invasion, tumor thrombus, pTN stage, WHO subtypes, gastrectomy, chemotherapy, and TIL. In univariate Cox regression analysis based on the data of the primary cohort, TIL, gastrectomy, and all clinicopathologic parameters were independent prognostic factors for OS (Table 3). Compared to the primary cohort, the age and tumor size were excluded in the validation cohort (Table 4). Moreover, pTN stage contained information on LN metastasis, which was excluded from the multivariate Cox regression model. TIL was an independent prognostic factor for OS in multivariate Cox regression analysis which based on the data of the primary cohort or the validation cohort (HR 0.431, 95% CI: 0.347–0.534, *P* < 0.001; HR 0.500, 95% CI: 0.313–0.797, *P* = 0.004, Tables 3, 4). The HR score for TIL was < 1, revealing that the high level of TIL had a protective effect on patient survival.

**Table 3:** Univariate and multivariate cox regression analyses of clinicopathological parameters and TIL in primary cohort.

**Clinicopathological parameters**	Univariate analysis^*^			Multivariate analysis^*^		
	**HR**	**95% CI**	***P-*value**	**HR**	**95% CI**	***P*-value**
Gender (male/female)	0.948	0.766–1.174	0.625			
Age ( ≤ 50/>50)	1.803	1.327–2.450	** < 0.001**	1.886	1.383–2.573	** < 0.001**
Tumor Size ( ≤ 5 cm/>5 cm)	2.505	2.042–3.074	** < 0.001**	1.320	1.058–1.647	**0.014**
Histological Grade (high/low)	2.561	2.058–3.187	** < 0.001**	1.208	0.924–1.581	0.167
LN metastatic (+/−)	5.572	4.234–7.332	** < 0.001**			
Nerve invasion (+/−)	3.015	2.454–3.704	** < 0.001**	1.342	1.071–1.683	**0.011**
Tumor Thrombus (+/−)	2.132	1.743–2.608	** < 0.001**	1.249	1.012–1.543	**0.039**
**pTN (I-III)**						
I	Reference			Reference		
II	4.691	3.009–7.313	** < 0.001**	2.973	1.860–4.753	** < 0.001**
III	12.63	8.313–19.18	** < 0.001**	5.952	3.675–9.638	** < 0.001**
**WHO SUBTYPES**						
Tubular	Reference			Reference		
Mucinous	1.439	1.058–1.957	**0.020**	0.568	0.407–0.792	**0.001**
Papillary	1.090	0.697–1.703	0.706	0.949	0.609–1.572	0.929
Poorly cohesive	1.471	1.131–1.913	**0.004**	0.761	0.574–1.009	0.057
Undifferentiated	2.810	2.008–3.933	** < 0.001**	1.569	1.101–2.237	**0.013**
Gastrectomy (Palliative/Radical)	3.333	2.558–4.343	** < 0.001**	2.060	1.567–2.710	** < 0.001**
Chemotherapy (+/−)	1.115	0.919–1.354	0.269			
TIL (high/low)	0.303	0.247–0.372	** < 0.001**	0.431	0.347–0.534	** < 0.001**

*Values in bold signify P < 0.05*.

* *The Univariate and Multivariate Cox Regression Analyses for the complete cohort was showed in Supplemental Table 5*.

**Table 4:** Univariate and multivariate cox regression analyses of clinicopathological parameters and TIL in validation cohort.

**Clinicopathological parameters**	**Univariate analysis**			**Multivariate analysis**		
	**HR**	**95% CI**	***P-*value**	**HR**	**95% CI**	***P*-value**
Gender (male/female)	0.716	0.486−1.056	0.092			
Age (≤ 50/>50)	1.151	0.616−2.152	0.660			
Tumor Size (≤ 5/>5 cm)	1.472	0.995−2.179	0.053			
Histological Grade (high/low)	2.522	1.663−3.826	**<0.001**	1.446	0.850−2.460	0.174
LN metastatic (+/−)	4.677	2.697−8.109	**<0.001**			
Nerve invasion (+/−)	2.504	1.632−3.841	**<0.001**	1.366	0.828−2.252	0.222
Tumor Thrombus (+/−)	2.867	1.824−4.508	**<0.001**	1.025	0.603−1.743	0.927
**pTN (I-III)**						
I	Reference			Reference		
II	4.809	1.851−12.50	**<0.001**	3.076	1.103−8.573	**0.032**
III	15.21	6.113−37.84	**<0.001**	7.458	2.583−21.54	**<0.001**
**WHO SUBTYPES**						
Tubular	Reference			Reference		
Mucinous	2.105	1.118−3.965	**0.021**	0.792	0.398−1.573	0.505
Papillary	2.187	0.990−4.835	0.053	2.464	0.967−6.279	0.059
Poorly cohesive	2.562	1.520−4.318	**<0.001**	1.023	0.565−1.851	0.940
Undifferentiated	2.051	1.132−3.717	**0.018**	1.042	0.539−2.016	0.902
Gastrectomy (Palliative/Radical)	6.421	3.695−11.16	**<0.001**	3.583	1.916−6.701	**<0.001**
Chemotherapy (+/−)	1.426	0.967−2.104	0.073			
TIL (high/low)	0.326	0.212−0.502	**<0.001**	0.500	0.313−0.797	**0.004**

*Values in bold signify P < 0.05*.

### Nomogram Development and Validation

**Figure 3:**
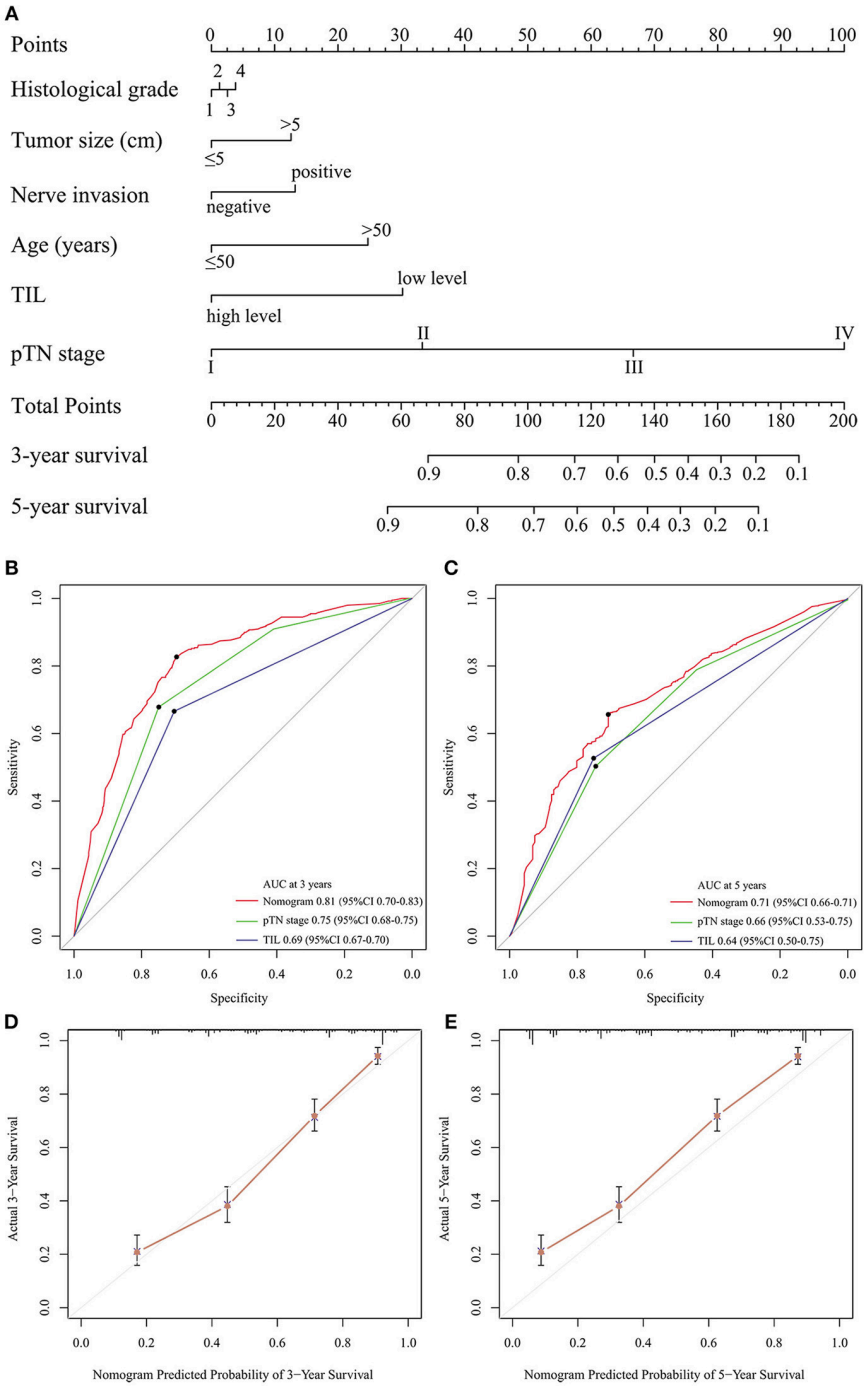
Evaluation of the integrated systemic nomogram in the primary cohort. To use the nomogram **(A)**, the value attributed to an individual patient is located on each variable axis, and an upwards line is drawn to determine the points received for each variable. The sum of these scores is located on the total points axis, and a downward line is drawn to the survival axis to determine the likelihood of 3- or 5-year survival. Time-dependent ROC curves by nomogram, pTN stage and TIL for 3-year **(B)** and 5-year **(C)** OS in GC patients. The calibration curve for predicting patient aurvival at 3-year **(D)**, and 5-year **(E)** in the primary cohort.

Backward stepwise selection with the Akaike information criterion (AIC) was used in building the Cox proportional hazards regression model to find a best-fit model among these independent factors. Finally, a nomogram that integrated six factors (histological grade, tumor size, nerve invasion, age, TIL, and pTN stage) was used to predict 3- and 5-year OS in the primary cohort (Figure 3A). The C-index of the nomogram was 0.774 (95% CI: 0.749–0.799), which was higher than that of pTN stage (0.717) and TIL (0.648). The time-dependent ROC curve showed a high sensitivity and specificity for predicting 3- and 5-year OS (Figures 3B,C). The calibration plot for the probability of surviving 3- or 5- years after surgery showed a good correlation between the prediction by the nomogram and the actual observation (Figures 3D,E).

The predictive accuracy of the nomogram for OS was validated. The designed nomogram was used to assess OS for the validation cohort. The C-index of the nomogram for predicting OS was 0.760 (95% CI: 0.704–0.815) in the validation cohort, which was also higher than that of pTN (0.720) and TIL (0.633). The ROC curve also showed similar results (Supplemental Figures 4A,B). The calibration curves showed good consistency in the probability of 3- and 5-year survival between the actual observation and the nomogram prediction (Supplemental Figures 4C,D). These results suggest that the nomogram is a more accurate and useful tool for the prediction of OS in patients with gastric cancer.

## Discussion

The immune system plays a pivotal role in tumor surveillance and host protection. Infiltrating lymphocytes, which are part of the host immune system response to internal or external pathogenic factors, can be seen in a variety of diseases. For breast cancer, there is an international standard of TIL scoring, which was stipulated by the 2014 International TILs Working Group on Breast Cancer (16). There is no current consensus on TIL evaluation in GC. The purpose of the current study was to assess TIL in the primary cohort and compare OS by TIL scoring, and then to validate these results in the validation cohort. After a series of analyses, we found that “score 5: the total of TIL” was the best index for TIL evaluation. These data may also inform future international TIL scoring for GC. In this study, the principal finding was that TIL^hi^ correlates with a low rate of cancer metastasis and better patient survival. Malignant tumor patients are treated according to standard therapeutic guidelines based on the TNM stage. Due to the development of immunotherapy, the tumor immunity, and treatment response had become more important. TNM staging systems cannot reflect the information of host immune system response. Our study showed that the combination of TIL and TNM stage can provide comprehensive prognostic information for GC patients. However, since TIL are heterogeneous and different components have different functions, the development of exact evaluations of this complex system are needed. To alleviate this, we constructed a nomogram model using our data. This nomogram predicted OS with a C-index 0.774 for accuracy, which indicated a better prediction of OS than pTN stage or TIL. The ROC also showed higher sensitivity and specificity for predicting 3- and 5-year OS compared with the pTN stage and TIL. Therefore, our nomogram is a reliable tool to predict survival in patients with GC and is helpful for making individualized treatment decisions. Although our nomogram demonstrated good predictive accuracy for survival, there are still several limitations. First, the nomogram was established based on the data from an individual institution in China. Second, our study was a retrospective study, and there may exist selection bias during retrospective data collection. Therefore, our results need to be further verified in a prospective, large-scale collaborative study.

Clinicopathologic parameters, such as lymph node metastasis (31), are widely used to evaluate the tumor malignancy, and prognosis of patients. The most commonly observed clinicopathologic parameters are age, tumor size, histological grade, LN metastasis, neural invasion, tumor thrombus, pTN stage, and WHO subtype. The current analyses showed that the level of TIL is a prognostic indicator that is as crucial as other clinicopathologic parameters. Negative correlations between TIL and clinicopathologic parameters, which was also observed between TIL and OS, indicated that TIL represent an antitumor microenvironment in GC. In univariate and multivariate Cox regression analysis, TIL was an independent prognostic factor. TIL are a histologic prognostic feature of potential value, but they are not currently part of tumor staging. Saldanha et al. (22) point out “the emergence of immune checkpoint inhibitors has fueled interest in TIL because these cells are the biological engine underpinning this therapy.” These data may also inform novel therapeutic approaches, such as adoptive immune therapies in GC (32–35). The hypothesis mentioned by Rosenberg et al. (36), i.e., that TIL only have an antitumor function when highly infiltrated into the TME, has been confirmed in this study. Through pathological morphological observations, we found that TIL have noticeable differences in quantity and structure in GC tissue. According to the results of this study and others, TIL should be an index of susceptibility to immunotherapy, and the future orientation of this study will focus on the understanding of the TIL subpopulations and their functional state as well as how this relates to the cancer immunity cycle.

## Author Contributions

DZ and WH were co-first author and contributed conception and design of the study. DZ, CW, YT, and YH organize the database. BX and LC performed the statistical analysis. DZ and WH wrote the first draft of the manuscript. JJ and QL were the study guarantors. All authors contributed to manuscript revision, read, and approved the submitted version.

### Conflict of Interest Statement

The authors declare that the research was conducted in the absence of any commercial or financial relationships that could be construed as a potential conflict of interest.

## Supplementary Material

Supplemental Table 1 The sub-group detail of TIL scores.

Supplemental Table 2 The consistency test for TIL.

Supplement Table 3 The details of primary cohort, validation cohort and complete cohort.

Supplemental Table 4 The correlation between TIL and clinicopathological parameters in the complete cohort.

Supplemental Table 5 Univariate and multivariate cox regression analyses of clinicopathological parameters and TIL in the complete cohort.

Supplemental Table 6 Multivariate cox regression analyses of TIL scores.

Supplemental Figure 1 The definition of IM in tumors. Gastric cancers invade normal tissues from mucosa to serosa. The yellow line indicates the depth of the most invasive cancer cell **(A,B)**. We define the area between the red lines as IM, and the distance between the red line and yellow line is one 100-fold power field (200-PF) **(A)**. Sometimes the cancer invades near the serosa. The distance between them is less than a 100-PF **(B)**. In this situation, the serous is defined as the boundary of the IM.

Supplemental Figure 2 The ROC curve analysis. The ROC curve analysis of the series of the TIL **(A)**. Comparing the AUC, the TIL-total score (score 5) was selected as the final score of the TIL. The ROC curve analysis of the TIL, str-TIL, and iTu-TIL **(B)**. The AUC of the TIL showed the higher than str-TIL and iTu-TIL.

Supplemental Figure 3 The Relationship among the iTu-TIL, str-TIL, CD3^+^ TIL, and TIL. The correlation analysis of the relationship between iTu-TIL and TIL **(A)**, str-TIL and TIL **(A)**, and the number of CD3^+^ TIL and TIL **(B)**. The scores of iTu-TIL, str-TIL, CD3^+^ TIL, and TIL were plotted and analyzed by correlation coefficient analyses.

Supplemental Figure 4 The validation for the nomogram. Time-dependent (ROC) curves by nomogram, pTN staging and TIL for 3-year **(A)** and 5-year **(B)** OS in the validation cohort. The calibration curve for predicting patient survival at 3-year **(C)** and 5-year **(D)** in the validation cohort.

## Abbreviations

TIL: tumor-infiltrating lymphocytes
TME: tumor microenvironment
GC: gastric cancer
iTu-TIL: intratumoral TIL
str-TIL: stromal TIL
CT: center of the tumor
IM: invasive margin
pTN: pathological tumor and lymph node
LN: lymph node
OS: overall survival
C-index: concordance index
AIC: akaike information criterion
ROC: receiver operating characteristics
AUC: area under the ROC curve.
