# Supplementary material for: Scoring System for Tumor-Infiltrating Lymphocytes and Its Prognostic Value for Gastric Cancer
Source: Front Immunol. 2019 Jan 29;10:71. doi: 10.3389/fimmu.2019.00071 (PMC6361780; doi:10.3389/fimmu.2019.00071)
Supplement: Supplemental Table 1 — The sub-group detail of TIL scores. [file Table_1.DOCX]

Supplemental Table 1. The Sub-Group Detail of TIL Scores

| TIL scores | cut-off value* | Primary cohort | | | | | | | | | Validation cohort | | | |
| --- | --- | --- | --- | --- | --- | --- | --- | --- | --- | --- | --- | --- | --- | --- |
|  |  | (*n*=833) | | | | | % | | | | (*n*=200) | | | % |
| Score 1: the intensity of TIL in CT | | | | |  | | | | |  | | |  |  |
| high | 2 and 3 | 259 | | | | | 31.1 | | | | 50 | | | 25.0 |
| low | 0 and 1 | 574 | | | | | 68.9 | | | | 150 | | | 75.0 |
| Score 2: the intensity of TIL in IM | | | | |  | | | | |  | | |  |  |
| high | 2 and 3 | 254 | | | | | 30.5 | | | | 56 | | | 28.0 |
| low | 0 and 1 | 579 | | | | | 69.5 | | | | 144 | | | 72.0 |
| Score 3: the TIL-ct region score | | | | |  | | | | |  | | |  |  |
| high | >0.35 | 352 | | | | | 42.3 | | | | 66 | | | 33.0 |
| low | ≤0.35 | 481 | | | | | 57.7 | | | | 134 | | | 67.0 |
| Score 4: the TIL-im region score | | |  | | |  | |  | | |  | | |  |
| high | >0.9 | 218 | | | | | 26.2 | | | | 52 | | | 26.0 |
| low | ≤0.9 | 615 | | | | | 73.8 | | | | 148 | | | 74.0 |
| Score 5: the TIL-total score | | | |  | | | | |  | | |  |  |  |
| high | >0.55 | 439 | | | | | 52.7 | | | | 93 | | | 46.5 |
| low | ≤0.55 | 394 | | | | | 47.3 | | | | 107 | | | 53.5 |

*The cut-off values were calculated by ROC curve analysis based on the date of the primary cohort.

The score 3: cut-off value was 0.35([AUC] 0.728, sensitivity 61.74%, specificity 76.90%);

The score 4: cut-off value was 0.90([AUC] 0.608, sensitivity 38.26%, specificity 85.71%);

The score 5: cut-off value was 0.55([AUC] 0.741, sensitivity 72.15%, specificity 66.43%).

The validation cohort was used the cut-off value calculated in the primary cohort to validate the analysis.
