# Supplementary material for: Scoring System for Tumor-Infiltrating Lymphocytes and Its Prognostic Value for Gastric Cancer
Source: Front Immunol. 2019 Jan 29;10:71. doi: 10.3389/fimmu.2019.00071 (PMC6361780; doi:10.3389/fimmu.2019.00071)
Supplement: Supplemental Table 2 — The consistency test for TIL. [file Table_2.DOCX]

Supplemental Table 2. The Consistency Test for TIL

|  | TIL | | *χ^2^* | *P* value | *κ* value | *P* value |
| --- | --- | --- | --- | --- | --- | --- |
|  | High | Low |  |  |  |  |
| iTu-TIL |  |  |  |  |  |  |
| Positive | 79 | 21 | 79.58 | **<0.001** | 0.630 | **<0.001** |
| Negative | 16 | 84 |  |  |  |  |
| str-TIL |  |  |  |  |  |  |
| Positive | 81 | 5 | 131.8 | **<0.001** | 0.809 | **<0.001** |
| Negative | 14 | 100 |  |  |  |  |
| CD3^+^ TIL |  |  |  |  |  |  |
| High | 39 | 4 | 27.94 | **<0.001** | 0.666 | **<0.001** |
| Low | 5 | 15 |  |  |  |  |

Values in bold signify *P*<0.05
