# Supplementary material for: Scoring System for Tumor-Infiltrating Lymphocytes and Its Prognostic Value for Gastric Cancer
Source: Front Immunol. 2019 Jan 29;10:71. doi: 10.3389/fimmu.2019.00071 (PMC6361780; doi:10.3389/fimmu.2019.00071)
Supplement: Supplement Table 3 — The details of primary cohort, validation cohort and complete cohort. [file Table_3.DOCX]

Supplement Table3. The Detials of Primary Cohort, Validation Cohort and Complete cohort..

| Characteristic | Primary cohort | | | Validation cohort | | Complete cohort | |
| --- | --- | --- | --- | --- | --- | --- | --- |
|  | (n=833) | % | (n=200) | | % | (n=1033) | % |
| Gender |  |  |  | |  |  |  |
| Male | 604 | 72.5 | 116 | | 58.0 | 720 | 69.7 |
| Female | 229 | 27.5 | 84 | | 42.0 | 313 | 30.3 |
| Age (year) |  |  |  | |  |  |  |
| ≤50 | 127 | 15.2 | 22 | | 11.0 | 149 | 14.4 |
| >50 | 706 | 84.8 | 178 | | 89.0 | 884 | 85.6 |
| Tumor size (cm) |  |  |  | |  |  |  |
| ≤5 | 396 | 47.5 | 99 | | 49.5 | 495 | 47.9 |
| >5 | 437 | 52.5 | 101 | | 50.5 | 538 | 52.1 |
| Histological grade |  |  |  | |  |  |  |
| Well | 41 | 4.9 | 10 | | 5.0 | 51 | 4.9 |
| Moderately | 307 | 36.9 | 72 | | 36.0 | 379 | 36.7 |
| Poor | 485 | 58.2 | 118 | | 59.0 | 603 | 58.4 |
| LN metastasis |  |  |  | |  |  |  |
| Positive | 520 | 62.4 | 128 | | 64.0 | 648 | 62.7 |
| Negative | 313 | 37.6 | 72 | | 36.0 | 385 | 37.3 |
| Neural invasion |  |  |  | |  |  |  |
| Positive | 413 | 49.6 | 112 | | 56.0 | 525 | 50.8 |
| Negative | 420 | 50.4 | 88 | | 44.0 | 508 | 49.2 |
| Tumor thrombus |  |  |  | |  |  |  |
| Positive | 214 | 25.7 | 117 | | 58.5 | 331 | 32.0 |
| Negative | 619 | 74.3 | 83 | | 41.5 | 702 | 68.0 |
| pTN stage |  |  |  | |  |  |  |
| I | 214 | 25.7 | 49 | | 24.5 | 263 | 25.5 |
| II | 240 | 28.8 | 65 | | 32.5 | 308 | 29.5 |
| III | 379 | 45.5 | 86 | | 43.0 | 465 | 45.0 |
| WHO subtypes |  |  |  | |  |  |  |
| Tubular | 526 | 63.1 | 123 | | 61.5 | 649 | 62.8 |
| Mucinous | 81 | 9.7 | 17 | | 8.5 | 98 | 9.5 |
| Papillary | 45 | 5.4 | 11 | | 5.5 | 56 | 5.4 |
| Poorly cohesive | 129 | 15.5 | 26 | | 13.0 | 155 | 15.0 |
| Undifferentiated | 52 | 6.2 | 23 | | 11.5 | 75 | 7.3 |
| Gastrectomy |  |  |  | |  |  |  |
| Radical | 761 | 91.4 | 184 | | 92.0 | 945 | 91.5 |
| Palliative | 72 | 8.3 | 16 | | 8.0 | 88 | 8.5 |
| Chemotherapy |  |  |  | |  |  |  |
| Positive | 327 | 39.3 | 85 | | 57.5 | 412 | 39.9 |
| Negative | 506 | 60.7 | 115 | | 42.5 | 621 | 60.1 |
| TIL |  |  |  | |  |  |  |
| High | 439 | 52.7 | 93 | | 46.5 | 532 | 51.5 |
| Low | 394 | 47.3 | 107 | | 53.5 | 501 | 48.5 |
