# Supplementary material for: Scoring System for Tumor-Infiltrating Lymphocytes and Its Prognostic Value for Gastric Cancer
Source: Front Immunol. 2019 Jan 29;10:71. doi: 10.3389/fimmu.2019.00071 (PMC6361780; doi:10.3389/fimmu.2019.00071)
Supplement: Supplemental Table 4 — The correlation between TIL and clinicopathological parameters in the complete cohort. [file Table_4.DOCX]

Supplemental Table 4. The Correlation between TIL and Clinicopathological Parameters in the complete cohort

| Clinicopathological  parameters | Patients | TIL | | *χ^2^* | *P* value |
| --- | --- | --- | --- | --- | --- |
|  | (n=1033) | High | Low |  |  |
| Gender |  |  |  |  |  |
| Male | 720 | 368 | 352 | 0.144 | 0.704 |
| Female | 313 | 164 | 149 |  |  |
| Age (year) |  |  |  |  |  |
| ≤50 | 149 | 82 | 67 | 0.87 | 0.351 |
| >50 | 884 | 450 | 434 |  |  |
| Tumor size (cm) |  |  |  |  |  |
| ≤5 | 495 | 286 | 209 | 14.994 | <**0.001** |
| >5 | 538 | 246 | 292 |  |  |
| Histological grade |  |  |  |  |  |
| Well | 51 | 37 | 14 | 54.184 | <**0.001** |
| Moderately | 379 | 242 | 137 |  |  |
| Poor | 603 | 253 | 350 |  |  |
| Lymphatic node metastasis | |  |  |  |  |
| Positive | 648 | 269 | 379 | 69.444 | <**0.001** |
| Negative | 385 | 263 | 122 |  |  |
| Neural invasion |  |  |  |  |  |
| Positive | 525 | 215 | 310 | 47.555 | <**0.001** |
| Negative | 508 | 317 | 191 |  |  |
| Tumor thrombus |  |  |  |  |  |
| Positive | 331 | 141 | 190 | 15.454 | <**0.001** |
| Negative | 702 | 391 | 311 |  |  |
| pTN stage |  |  |  |  |  |
| I | 263 | 192 | 71 | 92.441 | <**0.001** |
| II | 305 | 170 | 135 |  |  |
| III | 465 | 170 | 295 |  |  |
| WHO subtypes |  |  |  |  |  |
| Tubular | 649 | 393 | 256 | 69.046 | <**0.001** |
| Mucinous | 98 | 26 | 72 |  |  |
| Papillary | 56 | 30 | 26 |  |  |
| Poorly cohesive | 155 | 60 | 95 |  |  |
| Undifferentiated | 75 | 23 | 52 |  |  |
| Gastrectomy |  |  |  |  |  |
| Radical | 945 | 505 | 440 | 16.692 | **<0.001** |
| Palliative | 88 | 27 | 61 |  |  |
| Chemotherapy |  |  |  |  |  |
| Positive | 412 | 194 | 218 | 5.344 | **0.021** |
| Negative | 621 | 338 | 283 |  |  |

Values in bold signify *P*<0.05
