# Supplementary material for: Scoring System for Tumor-Infiltrating Lymphocytes and Its Prognostic Value for Gastric Cancer
Source: Front Immunol. 2019 Jan 29;10:71. doi: 10.3389/fimmu.2019.00071 (PMC6361780; doi:10.3389/fimmu.2019.00071)
Supplement: Supplemental Table 5 — Univariate and multivariate cox regression analyses of clinicopathological parameters and TIL in the complete cohort. [file Table_5.DOCX]

Supplemental Table 5. Univariate and Multivariate Cox Regression Analyses of Clinicopathological Parameters and TILs

| Clinicopathological | Univariate analysis | | | Multivariate analysis | | |
| --- | --- | --- | --- | --- | --- | --- |
| parameters | *HR* | *95% CI* | *P* value | *HR* | *95% CI* | *P* value |
| Gender (male/female) | 0.899 | 0.748-1080 | 0.256 |  |  |  |
| Age (≤50/>50) | 1.658 | 1.259-2.184 | <**0.001** | 1.888 | 1.430-2.494 | <**0.001** |
| Tumor Size (≤5 cm/>5 cm) | 2.239 | 1.869-2.682 | <**0.001** | 1.191 | 0.982-1.444 | 0.076 |
| Histological Grade (high/low) | 2.556 | 2.107-3.102 | <**0.001** | 1.284 | 1.015-1.625 | **0.037** |
| LN metastatic (+/-) | 5.369 | 4.200-6.864 | <**0.001** |  |  |  |
| Nerve invasion (+/-) | 2.887 | 2.399-3.474 | <**0.001** | 1.300 | 1.062-1.593 | **0.011** |
| Tumor Thrombus (+/-) | 2.054 | 1.727-2.444 | <**0.001** | 1.146 | 0.956-1.373 | 0.141 |
| pTN (I-III) |  |  |  |  |  |  |
| I | Reference | |  | Reference | |  |
| II | 4.687 | 3.134-7.009 | <**0.001** | 3.061 | 1.999-4.686 | <**0.001** |
| III | 13.07 | 8.943-19.10 | <**0.001** | 6.584 | 4.259-10.18 | <**0.001** |
| WHO subtypes |  |  |  |  |  |  |
| Tubular | Reference | |  | Reference | |  |
| Mucinous | 1.547 | 1.173-2.040 | **0.002** | 0.601 | 0.446-0.810 | **0.001** |
| Papillary | 1.255 | 0.851-1.850 | 0.252 | 1.141 | 0.751-1.733 | 0.537 |
| Poorly cohesive | 1.637 | 1.296-2.070 | <**0.001** | 0.843 | 0.657-1.081 | 0.179 |
| Undifferentiated | 2.459 | 1.837-3.291 | <**0.001** | 1.369 | 1.008-1.859 | **0.045** |
| Gastrectomy (Palliative/Radical) | 3.708 | 2.923-4.705 | <**0.001** | 2.319 | 1.811-2.968 | <**0.001** |
| Chemotherapy (+/-) | 1.146 | 0.964-1.363 | 0.122 |  |  |  |
| TIL (high/low) | 0.311 | 0.258-0.373 | <**0.001** | 0.440 | 0.362-0.534 | <**0.001** |

Values in bold signify *P*<0.05
