# Supplementary material for: Scoring System for Tumor-Infiltrating Lymphocytes and Its Prognostic Value for Gastric Cancer
Source: Front Immunol. 2019 Jan 29;10:71. doi: 10.3389/fimmu.2019.00071 (PMC6361780; doi:10.3389/fimmu.2019.00071)
Supplement: Supplemental Table 6 — Multivariate cox regression analyses of TIL scores. [file Table_6.DOCX]

Supplemental Table 6. Multivariate Cox Regression Analyses of TIL scores

| Clinicopathological | Multivariate Cox analysis* | | | Multivariate Cox analysis** | | |
| --- | --- | --- | --- | --- | --- | --- |
| parameters | *HR* | *95% CI* | *P* value | *HR* | *95% CI* | *P* value |
| Age (≤50/>50) | 1.817 | 1.333-2.477 | <**0.001** | - | - | **-** |
| Tumor Size (≤5 cm/>5 cm) | 1.321 | 1.058-1.648 | **0.014** | - | - | - |
| Histological Grade (high/low) | 1.371 | 1.049-1.792 | **0.021** | 1.726 | 1.030-2.894 | **0.038** |
| Nerve invasion (+/-) | 1.440 | 1.150-1.803 | **0.001** | 1.325 | 0.803-2.187 | 0.271 |
| Tumor Thrombus (+/-) | 1.281 | 1.039-1.580 | **0.021** | 1.100 | 0.648-1.869 | 0.723 |
| pTN (I-III) |  |  |  |  |  |  |
| I | Reference | |  |  |  |  |
| II | 2.734 | 1.711-4.370 | <**0.001** | 3.204 | 1.147-8.950 | **0.026** |
| III | 5.272 | 3.268-8.506 | <**0.001** | 8.095 | 2.805-23.36 | <**0.001** |
| WHO subtypes |  |  |  |  |  |  |
| Tubular | Reference | |  |  |  |  |
| Mucinous | 0.611 | 0.440-0.848 | **0.003** | 0.913 | 0.459-1.816 | 0.794 |
| Papillary | 1.108 | 0.691-1.778 | 0.671 | 3.068 | 1.199-7.853 | **0.019** |
| Poorly cohesive | 0.763 | 0.575-1.014 | 0.062 | 1.042 | 0.577-1.883 | 0.892 |
| Undifferentiated | 1.393 | 0.975-1.989 | 0.069 | 0.918 | 0.470-1.792 | 0.802 |
| Gastrectomy (Palliative/Radical) | 1.883 | 1.429-2.480 | <**0.001** | 3.800 | 2.049-7.050 | <**0.001** |
| Score 1 (high/low) | 0.346 | 0.256-0.468 | <**0.001** | 0.961 | 0.591-1.561 | 0.871 |

Values in bold signify *P* <0.05

| Clinicopathological | Multivariate Cox analysis* | | | Multivariate Cox analysis** | | |
| --- | --- | --- | --- | --- | --- | --- |
| parameters | *HR* | *95% CI* | *P* value | *HR* | *95% CI* | *P* value |
| Age (≤50/>50) | 1.905 | 1.397-2.598 | <**0.001** | - | - | **-** |
| Tumor Size (≤5 cm/>5 cm) | 1.377 | 1.101-1.723 | **0.005** | - | - | - |
| Histological Grade (high/low) | 1.267 | 0.968-1.658 | 0.085 | 1.507 | 0.883-2.573 | 0.133 |
| Nerve invasion (+/-) | 1.297 | 1.033-1.629 | **0.025** | 1.324 | 0.803-2.182 | 0.271 |
| Tumor Thrombus (+/-) | 1.291 | 1.046-1.594 | **0.017** | 1.127 | 0.661-1.922 | 0.660 |
| pTN (I-III) |  |  |  |  |  |  |
| I | Reference | |  |  |  |  |
| II | 3.457 | 2.153-5.550 | <**0.001** | 3.394 | 1.208-9.535 | **0.020** |
| III | 7.158 | 4.407-11.63 | <**0.001** | 8.387 | 2.869-24.52 | <**0.001** |
| WHO subtypes |  |  |  |  |  |  |
| Tubular | Reference | |  |  |  |  |
| Mucinous | 0.624 | 0.449-0.867 | **0.005** | 0.841 | 0.424-1.668 | 0.620 |
| Papillary | 1.058 | 0.660-1.695 | 0.816 | 2.416 | 0.937-6.234 | 0.068 |
| Poorly cohesive | 0.816 | 0.617-1.079 | 0.154 | 1.028 | 0.567-1.862 | 0.928 |
| Undifferentiated | 1.610 | 1.131-2.292 | **0.008** | 0.955 | 0.490-1.859 | 0.892 |
| Gastrectomy (Palliative/Radical) | 1.941 | 1.475-2.553 | <**0.001** | 3.740 | 2.006-6.975 | <**0.001** |
| Score 2 (high/low) | 0.415 | 0.322-0.535 | <**0.001** | 0.570 | 0.340-0.954 | **0.033** |

Values in bold signify *P* <0.05

| Clinicopathological | Multivariate Cox analysis* | | | Multivariate Cox analysis** | | |
| --- | --- | --- | --- | --- | --- | --- |
| parameters | *HR* | *95% CI* | *P* value | *HR* | *95% CI* | *P* value |
| Age (≤50/>50) | 1.813 | 1.330-2.471 | <**0.001** | - | - | **-** |
| Tumor Size (≤5 cm/>5 cm) | 1.322 | 1.060-1.649 | **0.013** | - | - | - |
| Histological Grade (high/low) | 1.264 | 0.967-1.652 | 0.087 | 1.688 | 1.006-2.830 | **0.047** |
| Nerve invasion (+/-) | 1.414 | 1.129-1.772 | **0.003** | 1.356 | 0.820-2.243 | 0.235 |
| Tumor Thrombus (+/-) | 1.230 | 0.998-1.517 | 0.053 | 1.055 | 0.618-1.801 | 0.845 |
| pTN (I-III) |  |  |  |  |  |  |
| I | Reference | |  |  |  |  |
| II | 2.769 | 1.734-4.422 | <**0.001** | 3.075 | 1.096-8.631 | **0.033** |
| III | 5.552 | 3.433-8.979 | <**0.001** | 7.864 | 2.721-22.73 | <**0.001** |
| WHO subtypes |  |  |  |  |  |  |
| Tubular | Reference | |  |  |  |  |
| Mucinous | 0.583 | 0.419-0.811 | **0.001** | 0.860 | 0.431-1.719 | 0.670 |
| Papillary | 0.998 | 0.621-1.604 | 0.993 | 2.827 | 1.101-7.259 | **0.031** |
| Poorly cohesive | 0.748 | 0.564-0.994 | **0.045** | 1.011 | 0.558-1.833 | 0.971 |
| Undifferentiated | 1.535 | 1.076-2.188 | **0.018** | 0.890 | 0.457-1.731 | 0.731 |
| Gastrectomy (Palliative/Radical) | 2.037 | 1.548-2.682 | <**0.001** | 3.817 | 2.051-7.104 | <**0.001** |
| Score 3 (high/low) | 0.430 | 0.339-0.546 | <**0.001** | 0.763 | 0.470-1.237 | 0.273 |

Values in bold signify *P* <0.05

| Clinicopathological | Multivariate Cox analysis* | | | Multivariate Cox analysis** | | |
| --- | --- | --- | --- | --- | --- | --- |
| parameters | *HR* | *95% CI* | *P* value | *HR* | *95% CI* | *P* value |
| Age (≤50/>50) | 1.879 | 1.377-2.563 | <**0.001** | - | - | **-** |
| Tumor Size (≤5 cm/>5 cm) | 1.404 | 1.122-1.757 | **0.003** | - | - | - |
| Histological Grade (high/low) | 1.224 | 0.934-1.602 | 0.143 | 1.543 | 0.907-2.623 | 0.109 |
| Nerve invasion (+/-) | 1.280 | 1.019-1.607 | **0.034** | 1.309 | 0.795-2.157 | 0.290 |
| Tumor Thrombus (+/-) | 1.283 | 1.039-1.584 | **0.020** | 1.119 | 0.656-1.909 | 0.680 |
| pTN (I-III) |  |  |  |  |  |  |
| I | Reference | |  |  |  |  |
| II | 3.498 | 2.178-5.618 | <**0.001** | 3.455 | 1.229-9.711 | **0.019** |
| III | 7.147 | 4.396-11.62 | <**0.001** | 8.504 | 2.914-24.82 | <**0.001** |
| WHO subtypes |  |  |  |  |  |  |
| Tubular | Reference | |  |  |  |  |
| Mucinous | 0.628 | 0.452-0.872 | **0.006** | 0.850 | 0.429-1.688 | 0.643 |
| Papillary | 1.002 | 0.624-1.609 | 0.993 | 2.477 | 0.961-6.387 | 0.060 |
| Poorly cohesive | 0.836 | 0.632-1.105 | 0.208 | 1.035 | 0.571-1.875 | 0.909 |
| Undifferentiated | 1.688 | 1.186-2.403 | **0.004** | 0.960 | 0.492-1.871 | 0.904 |
| Gastrectomy (Palliative/Radical) | 1.924 | 1.464-2.529 | <**0.001** | 3.772 | 2.023-7.035 | <**0.001** |
| Score 4 (high/low) | 0.352 | 0.265-0.468 | <**0.001** | 0.588 | 0.349-0.991 | **0.046** |

Values in bold signify *P* <0.05

| Clinicopathological | Multivariate Cox analysis* | | | Multivariate Cox analysis** | | |
| --- | --- | --- | --- | --- | --- | --- |
| parameters | *HR* | *95% CI* | *P* value | *HR* | *95% CI* | *P* value |
| Age (≤50/>50) | 1.886 | 1.383-2.573 | <**0.001** | - | - | **-** |
| Tumor Size (≤5 cm/>5 cm) | 1.320 | 1.058-1.647 | **0.014** | - | - | - |
| Histological Grade (high/low) | 1.208 | 0.924-1.581 | 0.167 | 1.446 | 0.850-2.460 | 0.174 |
| Nerve invasion (+/-) | 1.342 | 1.071-1.683 | **0.011** | 1.366 | 0.828-2.252 | 0.222 |
| Tumor Thrombus (+/-) | 1.249 | 1.012-1.543 | **0.039** | 1.025 | 0.603-1.743 | 0.927 |
| pTN (I-III) |  |  |  |  |  |  |
| I | Reference | |  |  |  |  |
| II | 2.973 | 1.860-4.753 | <**0.001** | 3.076 | 1.103-8.573 | **0.032** |
| III | 5.952 | 3.675-9.638 | <**0.001** | 7.458 | 2.583-21.54 | <**0.001** |
| WHO subtypes |  |  |  |  |  |  |
| Tubular | Reference | |  |  |  |  |
| Mucinous | 0.568 | 0.407-0.792 | **0.001** | 0.792 | 0.398-1.573 | 0.505 |
| Papillary | 0.949 | 0.609-1.572 | 0.929 | 2.464 | 0.967-6.279 | 0.059 |
| Poorly cohesive | 0.761 | 0.574-1.009 | 0.057 | 1.023 | 0.565-1.851 | 0.940 |
| Undifferentiated | 1.569 | 1.101-2.237 | **0.013** | 1.042 | 0.539-2.016 | 0.902 |
| Gastrectomy (Palliative/Radical) | 2.060 | 1.567-2.710 | <**0.001** | 3.583 | 1.916-6.701 | <**0.001** |
| Score 5 (high/low) | 0.431 | 0.347-0.534 | <**0.001** | 0.500 | 0.313-0.797 | **0.004** |

Values in bold signify *P* <0.05

* The multivariate Cox analysis was based on the data of the primary cohort.

** The multivariate Cox analysis was based on the data of the validation cohort.
