# Supplementary figures and images for: Scoring System for Tumor-Infiltrating Lymphocytes and Its Prognostic Value for Gastric Cancer
Source: Front Immunol. 2019 Jan 29;10:71. doi: 10.3389/fimmu.2019.00071 (PMC6361780; doi:10.3389/fimmu.2019.00071)

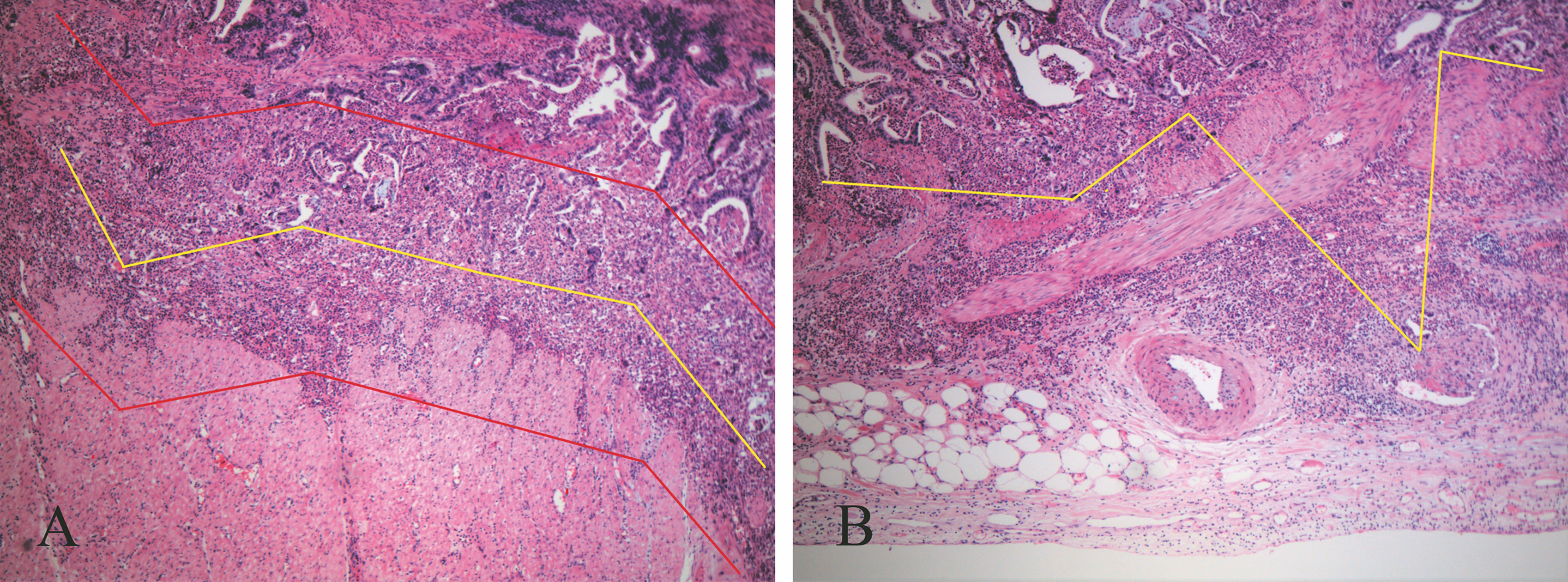

Supplement: Supplemental Figure 1 — The definition of IM in tumors. Gastric cancers invade normal tissues from mucosa to serosa. The yellow line indicates the depth of the most invasive cancer cell (A,B). We define the area between the red lines as IM, and the distance between the red line and yellow line is one 100-fold power field (200-PF) (A). Sometimes the cancer invades near the serosa. The distance between them is less than a 100-PF (B). In this situation, the serous is defined as the boundary of the IM. [file Image_1.TIF]

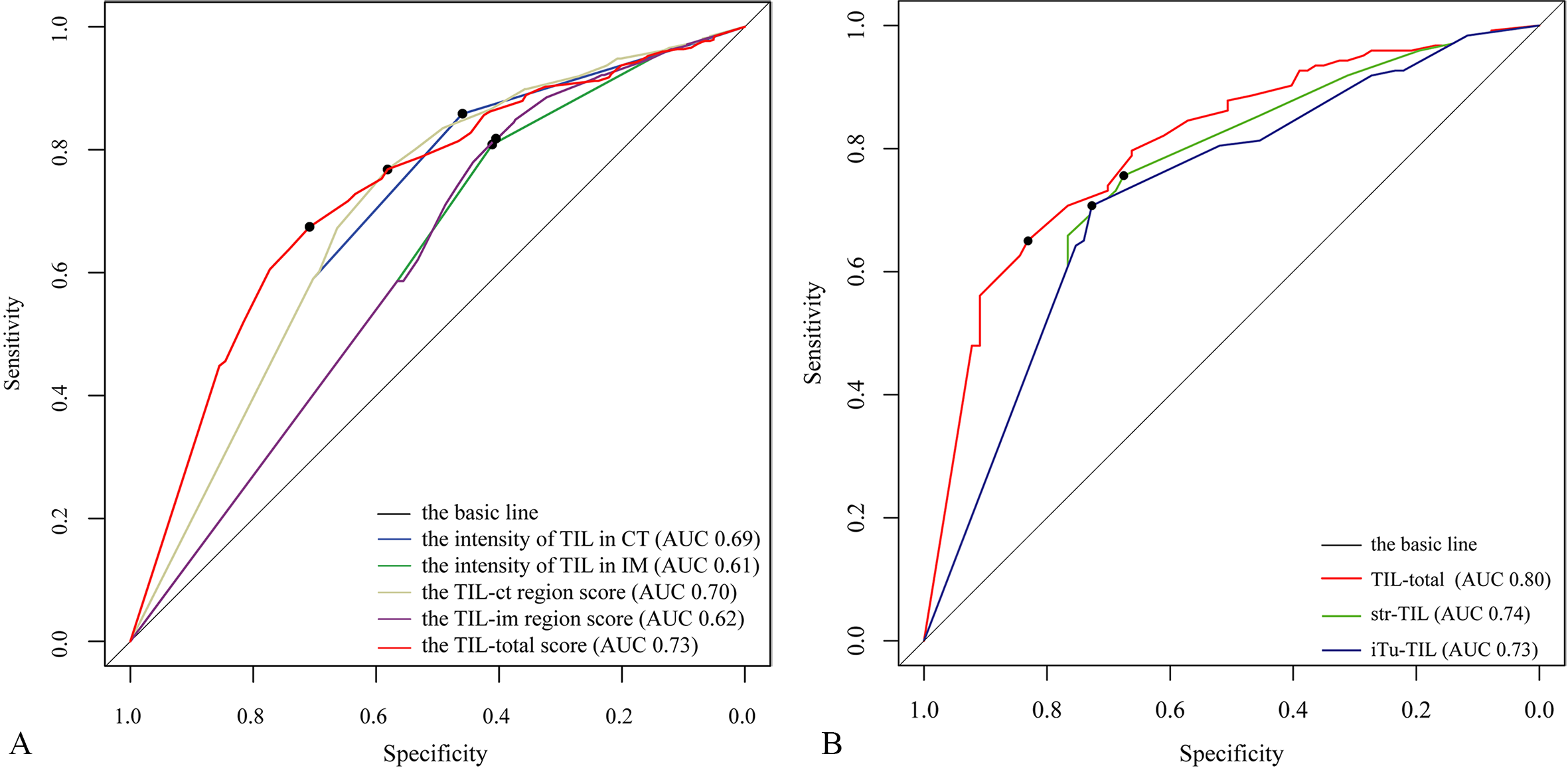

Supplement: Supplemental Figure 2 — The ROC curve analysis. The ROC curve analysis of the series of the TIL (A). Comparing the AUC, the TIL-total score (score 5) was selected as the final score of the TIL. The ROC curve analysis of the TIL, str-TIL, and iTu-TIL (B). The AUC of the TIL showed the higher than str-TIL and iTu-TIL. [file Image_2.TIF]

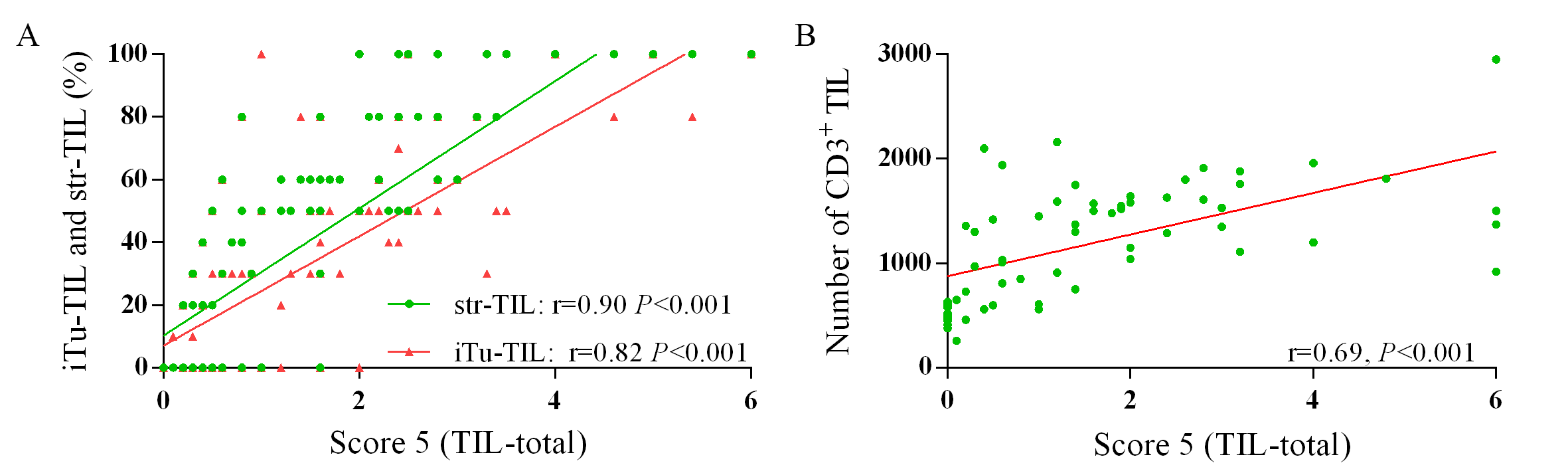

Supplement: Supplemental Figure 3 — The Relationship among the iTu-TIL, str-TIL, CD3+ TIL, and TIL. The correlation analysis of the relationship between iTu-TIL and TIL (A), str-TIL and TIL (A), and the number of CD3+ TIL and TIL (B). The scores of iTu-TIL, str-TIL, CD3+ TIL, and TIL were plotted and analyzed by correlation coefficient analyses. [file Image_3.TIF]

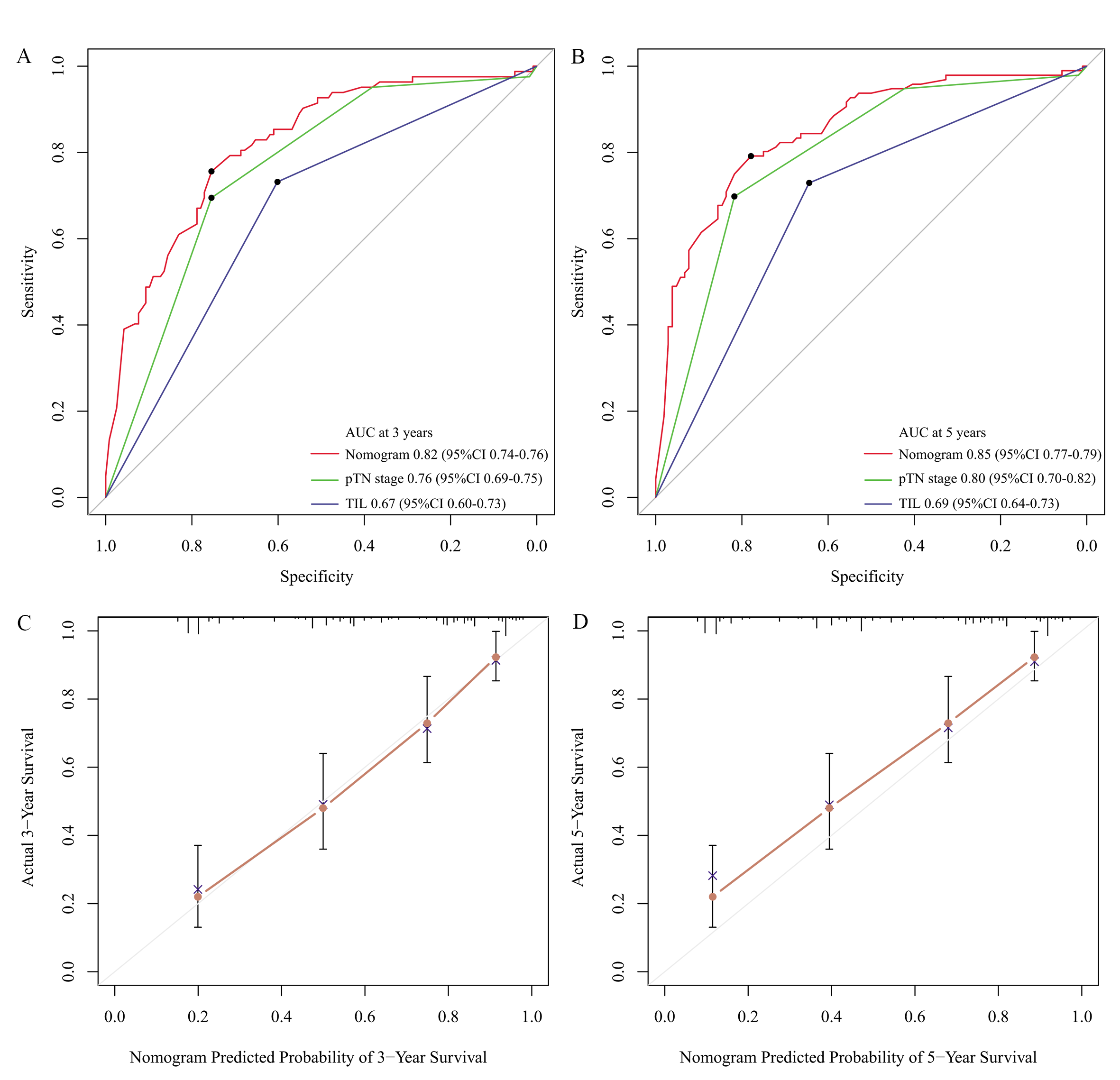

Supplement: Supplemental Figure 4 — The validation for the nomogram. Time-dependent (ROC) curves by nomogram, pTN staging and TIL for 3-year (A) and 5-year (B) OS in the validation cohort. The calibration curve for predicting patient survival at 3-year (C) and 5-year (D) in the validation cohort. [file Image_4.tif]
